# PyConvU-Net: a lightweight and multiscale network for biomedical image segmentation

**DOI:** 10.1186/s12859-020-03943-2

**Published:** 2021-01-07

**Authors:** Changyong Li, Yongxian Fan, Xiaodong Cai

**Affiliations:** School of Computer Science and Information Security, Guilin University of Electronic Technology, Guilin, 541004 China

**Keywords:** Biomedical image segmentation, Lightweight and multiscale network, PyConvU-net

## Abstract

**Background:**

With the development of deep learning (DL), more and more methods based on deep learning are proposed and achieve state-of-the-art performance in biomedical image segmentation. However, these methods are usually complex and require the support of powerful computing resources. According to the actual situation, it is impractical that we use huge computing resources in clinical situations. Thus, it is significant to develop accurate DL based biomedical image segmentation methods which depend on resources-constraint computing.

**Results:**

A lightweight and multiscale network called PyConvU-Net is proposed to potentially work with low-resources computing. Through strictly controlled experiments, PyConvU-Net predictions have a good performance on three biomedical image segmentation tasks with the fewest parameters.

**Conclusions:**

Our experimental results preliminarily demonstrate the potential of proposed PyConvU-Net in biomedical image segmentation with resources-constraint computing.

## Background

Biomedical image segmentation is typically the first critical step for biomedical image analysis [1]. Based on the accurate segmentation, multiple biological or medical analyses [2] can be performed subsequently, including cell counting [3], quantitative measurement of anatomical structure [4], cell phenotype analysis [5], subcellular localization [6], etc., providing valuable diagnostic information for doctors and researchers [7]. Although conventional image processing techniques are still employed for this time and labor-consuming task, they often cannot achieve the optimized performance due to different reasons, such as the limited capability of dealing with diverse images [8], lack of computing source, and so on.

With the rapid developments of DL based techniques, multiple researchers begin to investigate the potential applications to employ DL in biomedical image segmentation. One of the most popular applications is the U-Net [9]. Since the U-Net architecture was proposed in 2015, more and more researchers choose it as the backbone for their models because of its excellent performances. Now, U-Net is widely applied in the field of biomedical image segmentation and derives many variants. Such as MultiResUNet [10], Attention U-Net [11], UNet++ [12], and so on. All these variants based on U-Net solve some problems that are produced by U-Net in its applications.

The U-Net is an encoder-decoder architecture [13] consisting of a contracting path and an expansive path. The former is down-sampling which increases the receptive field [14] to gain more features. The latter recovers the feature extracted in the former and concatenates the corresponding feature map in the contracting path. The concatenation called skip connection [15] is an important part of U-Net because it combines the information in the architecture. But the way of getting context information in the U-Net is not capable of extracting more fine information to achieve better performance. To address the above problems, we chose a new convolution called pyramidal convolution [16] to get more information and to improve the performance of our model.

**Fig. 1:**
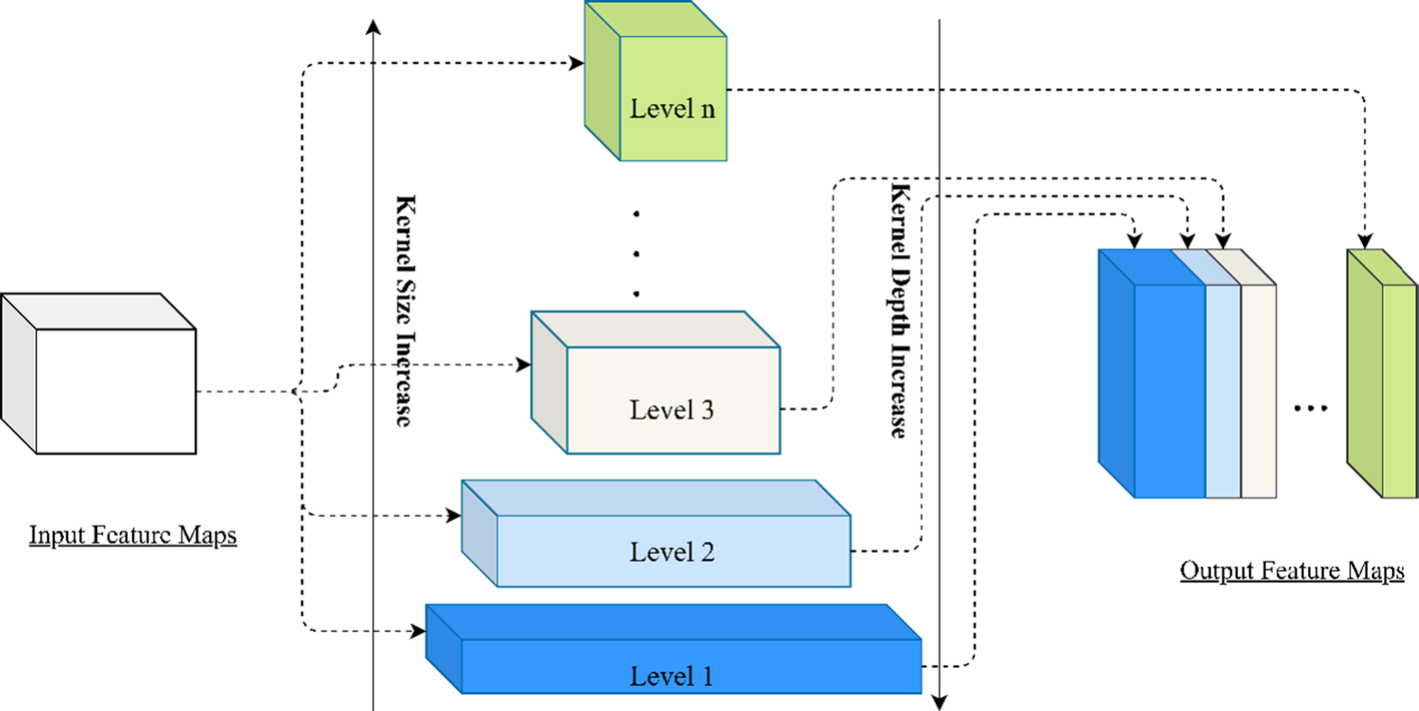
The structure of pyramidal convolution

The pyramidal convolution (PyConv) can process the input at multiple filter scales. It is illustrated in Fig. 1, contains a pyramid with *n* levels of different types of kernels. The goal of PyConv is to process the input at different kernel scales without increasing the computational cost or the model complexity (in terms of parameters). At each level of the PyConv, the kernel contains a different spatial size, increasing kernel size from the bottom of the pyramid to the top. Simultaneously with increasing the spatial size, the depth of the kernel is decreased from level 1 to level *n*. It involves different types of filters with varying sizes and depth so that it can capture different levels of details in the scene. Meanwhile, PyConv is also efficient and it does not increase the computational cost and parameters compared to standard convolution. Moreover, it is very flexible and extensible, providing a large space of potential network architectures for different applications.

In this paper, we develop a novel architecture called PyConvU-Net, an enhanced version of U-Net, demonstrating the implementation of PyConv in a standard U-Net architecture and applying it to biomedical images segmentation. We also compare the PyConvU-Net with many other models in different datasets, achieving a good performance while it has fewer number of parameters that can save computing power.

U-Net consists of a contracting path to capture context and a symmetric expanding path that enables precise localization. The contracting path follows the typical architecture of a convolutional network. It consists of the repeated application of two 3 × 3 convolutions (unpadded convolutions), each followed by a rectified linear unit (ReLU) [17] and a 2 × 2 max pooling operation with stride 2 for down-sampling. Every step in the expansive path consists of an up-sampling of the feature map followed by a 2 × 2 convolution (“up-convolution”) that halves the number of feature channels, a concatenation with the correspondingly cropped feature map from the contracting path, and two 3 × 3 convolutions, each followed by a ReLU. The cropping is necessary due to the loss of border pixels in every convolution. At the final layer, a 1 × 1 convolution is used to map each 64-component feature vector to the desired number of classes. In total the network has 23 convolutional layers.

The exploration of U-Net architecture has been a part of biomedical image segmentation research since its initial discovery. Many researchers propose a lot of variants of U-Net and continuously improve the performance of the structure. For example, MultiResUNet [10] combines the MutiRes module and U-Net, where MutiRes is an extension of residual connection [18]. In this module, three 3 × 3 convolution results are spliced together as a combined feature map, which is then added to the input feature after 1 × 1 convolution. Besides the MultiRes module, MultiResUNet has a significant part that is ResPath, the function of which is doing some additional convolution operations before the feature of the encoder are spliced with the corresponding features in the decoder. Another excellent network is Attention U-Net [11] that brings the attention mechanism into U-Net. Before stitching the feature at each resolution of the encoder and the corresponding feature in the decoder, an attention module that generates a gating signal to control the importance of the feature at a different spatial location is used to readjust the output characteristic of the encoder. The attention module combines ReLU and Sigmoid through 1 × 1x1 convolution to generate a weight map ${\upalpha }$ that can be corrected by multiplying the features in the encoder. UNet++ [12] also is a good architecture, starts with an encoder sub-network or backbone followed by a decoder sub-network. What distinguishes UNet++ from U-Net is the re-designed skip pathway that connects the two sub-networks and the use of deep supervision.

Besides the networks based on U-Net, there are also many segmentation networks for biomedical images. We choose a network called FCN [19] to compare with ours. FCN also is a good network for semantic segmentation. The reason why the network called FCN is because it converts the fully connected layers in traditional CNN [20] into convolutional layers. It is a fully convolutional network without a fully connected layer and can adapt to any size input. Besides, it makes use of a deconvolutional layer to increase the data size to achieve a better fine output result. What's more, it utilizes the skip connection to integrate the information in the different depth layers due to ensuring robustness and accuracy.

## Results

As shown in Table 1, we demonstrate the application of the PyConvU-Net to three different segmentation tasks. The first task is the segmentation of the lung in the CT images [21]. The dataset called kaggleLung which is provided by the Finding and Measuring Lungs in CT Data in Kaggle is a collection of 512 × 512 CT images, manually segmented lungs, and measurements in 2/3D, containing 267 2D images. We just choose the 2D images and split the dataset into two parts, of which the training set accounts for 80%, and the test set accounts for 20%. Each image comes with a corresponding fully annotated ground truth segmentation map for the lung (white) and other parts (black). The second dataset is similar to the first, except that the organ is replaced with the liver. Meanwhile, the liver dataset has 400 512 × 512 images more than kaggleLung. The above two datasets have the same challenges that images have an unclear edge and organs from different people have some slight differences. These challenges will affect the edge extract and location of organs we want to segment. The last dataset is ISBICell [22] is provided by the EM segmentation challenge that was started at ISBI 2012 and is still open for new contributions. The training data is a set of 30 512 × 512 images from serial section transmission electron microscopy of the Drosophila first instar larva ventral nerve cord (VNC) [23]. ISBICell has more detailed information (complex cell boundaries), which will test the model’s ability to handle details. Considering that these datasets have fewer samples, we have adopted some simple data augmentation methods to expand the datasets. These methods include horizontal flip, vertical flip, 90° rotation, and 180° rotation.

**Table 1:** The image segmentation datasets used in our experiments

Dataset	Images	Input size	Modality
kaggleLung	267	512 × 512	CT
liver	400	512 × 512	CT
ISBICell	30	512 × 512	Microscopy

**Fig. 2:**
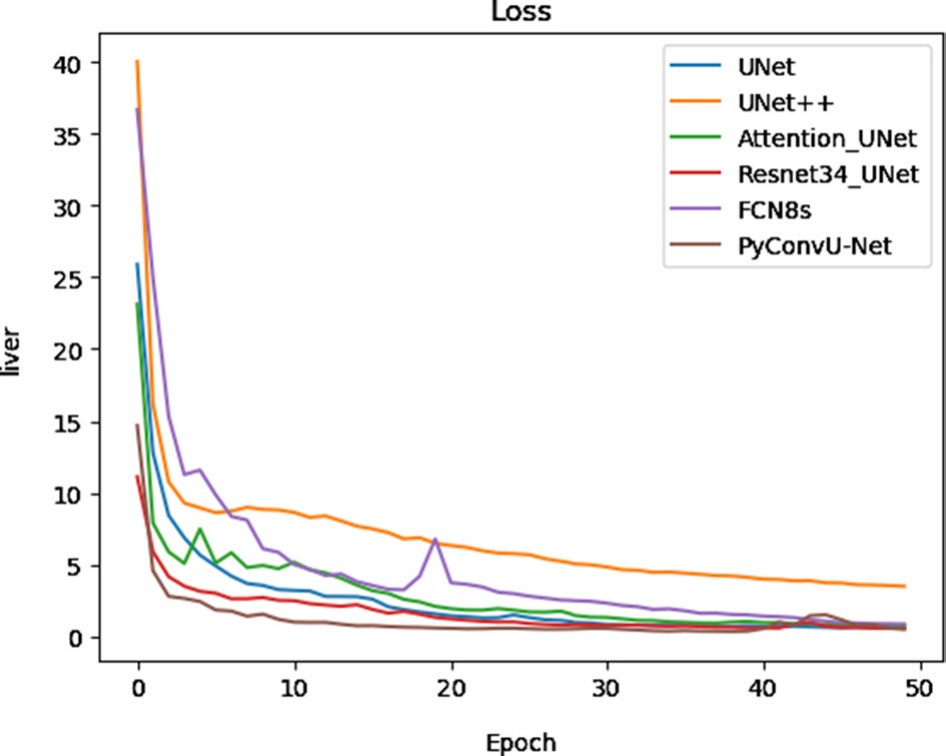
Training losses of different models

For comparison, we use FCN [19], the original U-Net, and a series of variants based on U-Net including UNet++, Resnet34_UNet, and Attention U-Net. First, the training losses of models are shown in Fig. 2. From Fig. 2, it is clear that the training losses of all models remain stable after the first 5 epochs training, only the loss of UNet++ is higher than other models after stable.

As shown in Table 2, we choose two metrics, MIoU [24] and Dice [25] respectively, to evaluate our model in the three segmentation tasks.

**Table 2:** MIoU and dice of different models in three datasets

	kaggleLung		liver		ISBICell	
	MIoU	Dice	MIoU	Dice	MIoU	Dice
U-Net	0.7279	0.7834	0.6207	0.7386	0.7742	0.8639
UNet++	0.6078	0.6471	0.6504	0.7690	0.7878	0.8808
Resnet34_UNet	0.9494	0.9721	0.6623	0.7451	**0.8398**	0.9115
Attention U-Net	0.7723	0.8278	0.6989	0.8083	0.8269	0.8945
FCN8s	0.9545	**0.9752**	0.5139	0.6447	0.8345	0.9005
PyConvU-Net	**0.9630**	0.9339	**0.7050**	**0.8227**	0.8385	**0.9117**

Bold numbers indicate the best performance

MIoU is to calculate the ratio of the intersection and union of the true value set and predicted value set, the formula is as follows.

1 $$MIoU = \frac{1}{k + 1}\mathop \sum \limits_{i = 0}^{k} \frac{TP}{{FN + FP + TP}}$$

where $\frac{TP}{{FN + FP + TP}}$ can be equivalent to the following formula.

2 $$\frac{TP}{{FN + FP + TP}} = \frac{{p_{ii} }}{{\mathop \sum \nolimits_{j = 0}^{k} p_{ij} + \mathop \sum \nolimits_{j = 0}^{k} p_{ji} - p_{ii} }}$$

where $k$ is the number of categories, $i$ represents the true value, $j$ represents the predicted value and $p_{ij}$ represents predicting $i$ as $j$. $p_{ii}$ is the number of true values.

Dice coefficient is a function that measures the similarity of two sets and is one of the commonly used evaluation indicators in semantic segmentation. The Dice coefficient is defined as the intersection of two times divided by the sum of pixels, which is similar to IoU, and its calculation formula is as follows.

3 $${\text{Dice}}\left( {{\text{X}},{\text{Y}}} \right) = \frac{{2\left| {X \cap Y} \right|}}{\left| X \right| + \left| Y \right|}$$

It is equivalent to the following formula.

4 $${\text{Dice}} = \frac{2TP}{{2TP + FP + FN}}$$

Our proposed method achieves the best performance in liver dataset and is much higher than in the second place. On the kaggleLung dataset, our proposed method does not get the first place but has a better performance than other models but U-Net. In the last segmentation task, PyConvU-Net performs similarly to other methods, without much prominence where it gets the champion evaluated by Dice and gets the second place evaluated by MIoU. In the experiments, we also measured the parameter size and computational complexity of different models respectively, listed in Table 3.

**Table 3:** Number of parameters and computational complexity of different networks

	U-Net	Unet++	Resnet34_UNet	Attention U-Net	FCN8s	PyConvU-Net
Number of parameters/MB	7.77	9.16	21.66	34.88	18.64	**3.7**
FLOPs/GMac	48.57	138.63	24.27	266.54	85.86	**10.65**

Bold numbers indicate the best performance

**Fig. 3:**
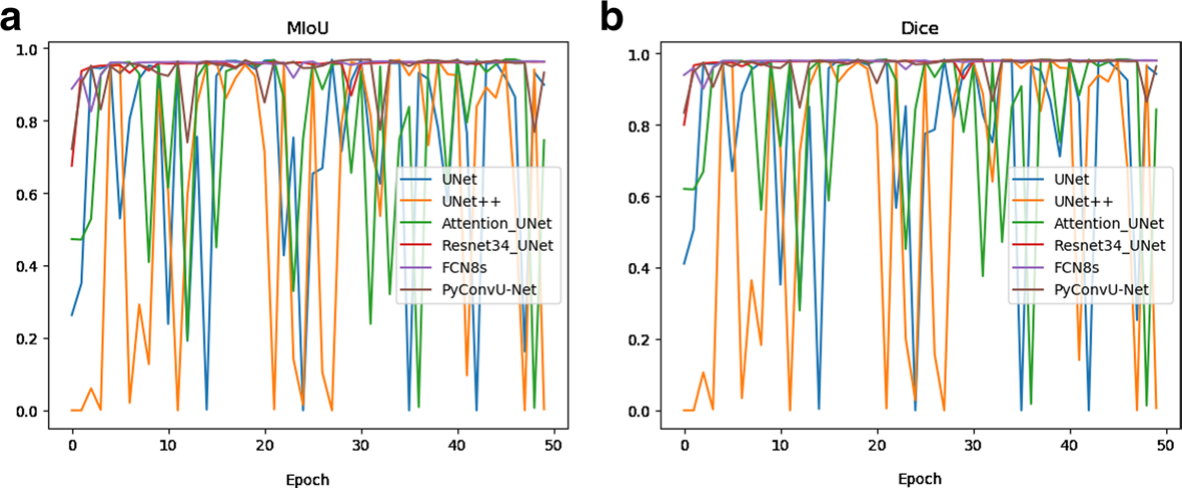
The evaluation of different models. **a** MIoU of different methods, **b** dice of different methods

From Fig. 3, the MIoU and Dice of our proposed method, FCN8s and Resnet34_UNet are stable after 3 epochs while can keep a high level. Other methods perform very unstably.

Our method has the fewest parameters which means our network does not need too much computational power. From this, we can see that even if we lose some precision in some aspect, we can keep the network lightweight while not affecting the segmentation tasks finished by our proposed model.

**Fig. 4:**
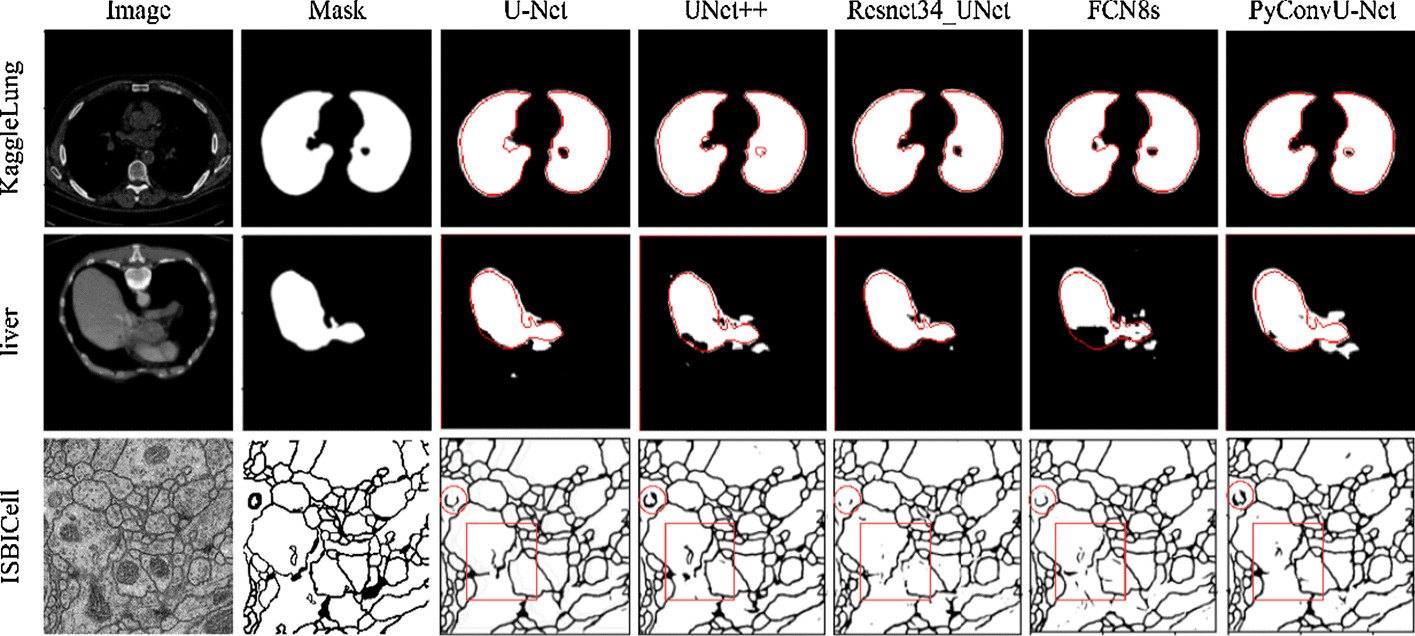
Segmentation comparisons. From left to right, the columns represent the original image, mask, U-Net predictions, U-Net++ predictions, Resnet34_UNet predictions, FCN8s predictions, and PyConvU-Net predictions respectively. The red curve shows the actual area of the organ. The markers of the last row indicate the key area

We put the predictions of different methods in Fig. 4.

All experiments were carried out in the PyTorch framework [26] and trained using Nvidia-RTX 2080Ti GPUs. These networks are trained for a total of 50 epochs and a batch size of 5.

## Discussion

Due to its excellent performance, U-Net is the most widely used backbone architecture for biomedical image segmentation in recent years. However, in our studies, we observe that U-Net will ignore detailed information when performing convolution operations [27]. We analyze this issue in detail and address it by proposing a lightweight and multiscale architecture PyConvU-Net which replaces the traditional convolution layer with the pyramidal convolution layer. This network which can extract multiple sequence feature information [28] not only achieves improvements in the biomedical image segmentation tasks [29] but also reduces the number of parameters.

We evaluate the proposed method on three biomedical image segmentation tasks. We can see from Table 2 that the proposed method does not outperform other methods on all datasets. The PyConvU-Net achieves first place on the liver dataset and much higher than the second place. However, it does not perform as well as FCN8s on the kaggleLung dataset, it just gets second in MIoU and third in Dice. In response to this phenomenon, we carefully consider the reasons for this phenomenon. We think the reason is that the liver dataset has a clear edge between different organs, however, the boundaries in the kaggleLung dataset are fuzzy. So the proposed method has shortcomings in the segmentation of images with blurred boundaries. This situation also happens in the ISBICell datasets. The cell images have many complex edges that are entangled with each other. To some extent, these boundaries are unclear, so PyConvU-Net does not have a very good performance on the ISBICell dataset. From the experimental results in Table 2, although the proposed model does not achieve the best performance on all tasks, it is still in a leading position. From the beginning, our goal is to minimize the number of model parameters and computational complexity without losing segmentation accuracy or losing the part of the accuracy. We list the number of parameters and the computational complexity of different models in Table 3. In terms of the number of parameters, U-Net has 7.77 MB parameters, our proposed model’s parameters are almost half U-Net’s. Meanwhile, in computational complexity, the metric is FLOPs. Our proposed model is far ahead in this regard.

Hence, the next step of our future work has three parts. One is improving the abilities to segment the image with blurred boundaries and edge extract to solve the problem of that loss of object edge. The second is to carry on reducing the number of parameters and computational complexity to implement model deployment on mobile devices. The last one is that we hope to achieve good performances in both segmentation accuracy and model lightweight and obtain an accurate and efficient biomedical image segmentation model.

## Conclusion

We propose a lightweight and multiscale network called PyConvU-Net which is constructed by pyramidal convolution based on U-Net. The purpose of pyramidal convolution is to utilize different size filters to specifically capture detailed information which is typically missed out in the traditional convolution. Through the exhaustive experiments and analysis, despite we use different kernel sizes, PyConvU-Net does not increase the number of parameters while maintaining good performance in different segmentation tasks. For future work, it will be interesting to explore improve the performance of our proposed architecture in other segmentation datasets.

## Methods

**Fig. 5:**
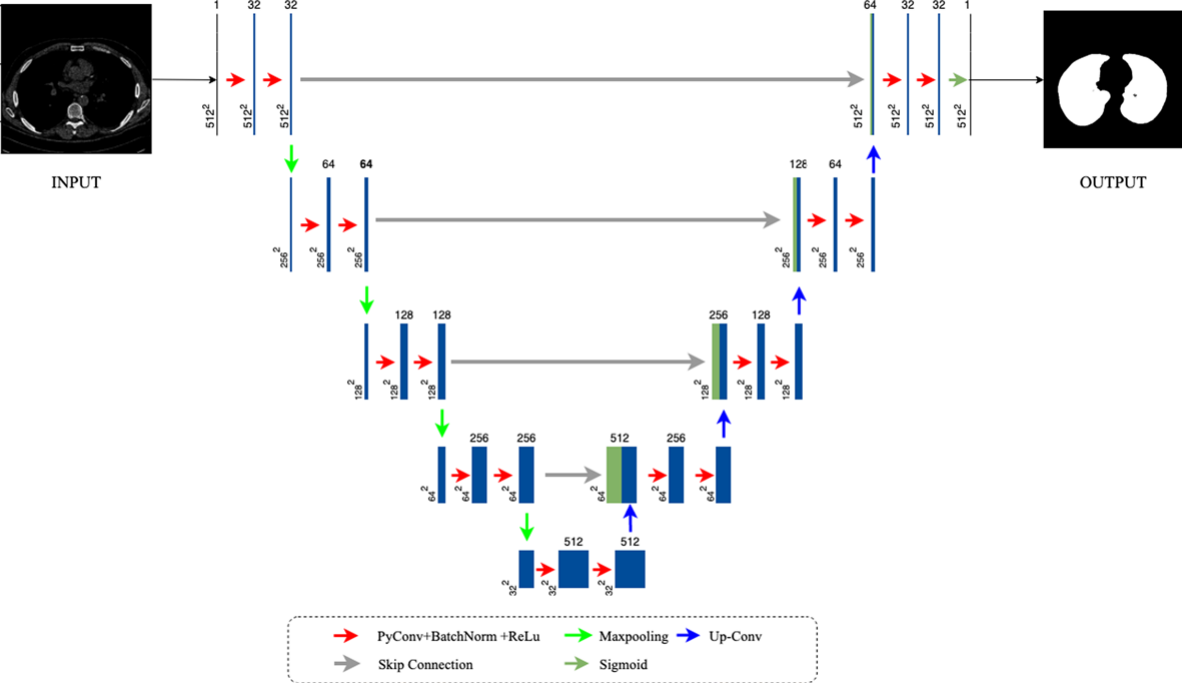
An overview of the proposed PyConvU-Net architecture

Figure 5 shows an overview of the suggested architecture. As seen, PyConvU-Net adopts a framework like U-Net's Encoder-Decoder. What distinguishes PyconvU-Net from U-Net is the re-designed convolutional layers (shown in red arrow) that replace the traditional convolution with the pyramidal convolution. As is shown in the legend which is at the bottom of Fig. 5, all convolution blocks are followed by a batch normalization layer [30] and a ReLU activation function.

Traditional convolutional using the fixed kernel size has entered a bottleneck period. It cannot gain more detailed information to improve the performance of the network. Therefore, we want to find another convolutional way that can extract as much as possible information in the biomedical images while not increasing the cost of computation. Pyramidal convolution came into our view at that time. We replace all conventional convolution layers in the U-Net with the pyramidal convolution. Also, we change the padding way in the U-Net. U-Net uses the valid padding that can reduce the size of the feature map after convolution, which can drop some fine information. To solve the problem, we change the valid padding into the same padding to ensure that the feature map does not change size before and after convolution. Meanwhile, At the final layer in the original U-Net, a 1 × 1 convolution is used to map each 64-component feature vector to the desired number of classes. However, the final layer in our proposed model is the Sigmoid activation function. This is because our mask image is a binary image. Through the Sigmoid activation function, the output of the network is a binary image that can be convenient to compare the difference between the two.

The number of parameters and FLOPs required for the standard convolution can be calculated by the following formulas:

5 $${\text{parameters}} = K_{1}^{2} \cdot FM_{i} \cdot FM_{o}$$

where $FM_{i}$ represents the input feature map, $FM_{o}$ represents the output feature map and $K_{1}$ is a spatial size of the kernel;

6 $${\text{FLOPs}} = K_{1}^{2} \cdot FM_{i} \cdot FM_{o} \cdot \left( {W \cdot H} \right)$$

where $W$ and $H$ represent the width and height of the output feature map respectively. However, in PyConv, for the input feature maps $FM_{i}$, each level of the PyConv $\left\{ {1, 2, 3, \cdots , n} \right\}$ applies different kernels with different spatial size for each level $\left\{ {K_{1}^{2} , K_{2}^{2} ,K_{3}^{2} , \cdots ,K_{n}^{2} } \right\}$ and with different kernel depths $\left\{ {FM_{i} ,\frac{{FM_{i} }}{{\left( {\frac{{K_{2}^{2} }}{{K_{1}^{2} }}} \right)}},\frac{{FM_{i} }}{{\left( {\frac{{K_{3}^{2} }}{{K_{1}^{2} }}} \right)}}, \cdots ,\frac{{FM_{i} }}{{\left( {\frac{{K_{n}^{2} }}{{K_{1}^{2} }}} \right)}}} \right\}$ (From Fig. 1, the kernel depth decreases as the kernel size increases). Afterwards, PyConv will output a different number of output feature maps $\left\{ {FM_{o1} ,FM_{o2} ,FM_{o3} , \cdots ,FM_{on} } \right\}$. Therefore, the number of parameters and FLOPs for PyConv are as follows:

7 $${\text{parameters}} = \mathop \sum \limits_{z = 1}^{n} K_{z}^{2} \cdot \frac{{FM_{i} }}{{\left( {\frac{{K_{z}^{2} }}{{K_{1}^{2} }}} \right)}} \cdot FM_{oz}$$

8 $${\text{FLOPs}} = \mathop \sum \limits_{z = 1}^{n} K_{z}^{2} \cdot \frac{{FM_{i} }}{{\left( {\frac{{K_{z}^{2} }}{{K_{1}^{2} }}} \right)}} \cdot FM_{oz} \cdot \left( {W \cdot H} \right)$$

where $FM_{o1} + FM_{o2} + FM_{o3} + \cdots + FM_{on} = FM_{o}$ and $K_{z}^{2} \cdot \frac{{FM_{i} }}{{\left( {\frac{{K_{z}^{2} }}{{K_{1}^{2} }}} \right)}}$ can be simplified as $K_{1}^{2} \cdot FM_{i}$. With Eqs. (7) and (8), regardless of the number of levels of PyConv and the increasing kernel size, the computational cost (in terms of FLOPs) and the number of parameters are the same as the standard convolution with a single kernel size.

According to the above analysis, the proposed model has two advantages. One is multiscale convolution. PyConvU-Net utilizes different kernel sizes to do convolution operations, which can gain more detailed information. The small size kernel focuses on details, capturing information about smaller objects, while the large size kernel provides more information about larger objects. The other is efficiency. Comparing with the U-Net, PyConvU-Net has a similar number of parameters and requirements in computational resources, as shown in Eqs. (7) and (8). Meanwhile, PyConvU-Net offers a high degree of parallelism due to the fact that the pyramid levels can be independently computed in parallel.

## Availability and requirements

- The kaggleLung dataset: https://www.kaggle.com/kmader/finding-lungs-in-ct-data
- The liver dataset: https://www.kaggle.com/stevenazy/liver-dataset
- The ISBICell dataset: http://brainiac2.mit.edu/isbi_challenge/home
- Project name: Biomedical image segmentation
- Project home page: https://github.com/StevenAZy/PyConvU-Net
- Operating systems: Ubuntu 18.04
- Programming language: Python3.7
- License: GNU GPL

## Abbreviations

CT: Computed tomography
DL: Deep learning
MIoU: Mean intersection over union
PyConv: Pyramidal convolution
ReLU: Rectified linear unit
VNC: Ventral nerve cord
FCN: Fully convolutional networks
FLOPs: Floating point operations
